# A retrospective analysis of the treatment results for advanced synchronous head and neck and esophageal cancer

**DOI:** 10.1259/bjro.20190015

**Published:** 2019-08-02

**Authors:** Kang-Hsing Fan, Yin-Kai Chao, Joseph Tung-Chieh Chang, Ng-Ming Tsang, Chun-Ta Liao, Kai-Ping Chang, Chien-Yu Lin, Hong-Ming Wang, Cheng-Lung Hsu, Shiang-Fu Huang

**Affiliations:** 1 Department of Radiation Oncology, Chang-Gung Memorial Hospital at Linkou; Chang Gung University College of Medicine,; 2 Division of Thoracic Surgery, Chang Gung Memorial Hospital, College of Medicine, Chang Gung University, Taoyuan, Taiwan; 3 Xiamen Chang Gung Memorial Hospital, Xiamen, Fujian, China; 4 Department of Otorhinolaryngology, Head and Neck Surgery, Chang-Gung Memorial Hospital at Linkou; Chang Gung University College of Medicine,; 5 Department of Medical Oncology, Chang-Gung Memorial Hospital at Linkou; Chang Gung University College of Medicine,

## Abstract

**Objective:**

The treatments for synchronous head and neck cancer (HNC) and esophageal cancer (ESC) are toxic and difficult to employ. The aim of this study was to identify the feasibility of a protracted, less toxic treatment course and prognostic factor of synchronous HNC and ESC.

**Methods:**

Cancer registry data from 2004 to 2012 were reviewed. The inclusion criteria were two cancer diagnoses within 30 days, and Stage III/IV HNC or Stage II–IV ESC that chemoradiation therapy was indicated. Evident metastasis, Eastern Cooperative Oncology Group performance score >2, a history of prior cancer, or palliative treatment were excluded. Survival rates and patient and treatment characteristics were analyzed.

**Results:**

There were 51 eligible cases. The 2 year overall survival rate was 25.1%. Univariate analysis found that anemia, larynx/hypopharynx HNC, and no esophagectomy correlated with poor overall survival. Multivariate analysis demonstrated that anemia and no esophagectomy were independent poor prognostic factors. The 2 year progression-free survival rate was 14.8%. Univariate analysis found only no esophagectomy correlated with poor progression-free survival.

**Conclusion:**

The outcomes are poor for patients with advanced synchronous HNC and ESC. Radiotherapy with a split or protracted course does not result in inferior treatment result and can be considered when the aim is to avoid adverse events. Esophagectomy correlated with good prognosis and should be performed for patients if possible.

**Advances in knowledge:**

The treatment results of synchronous HNC and ESC is poor. A protracted chemoradiation course for synchronous HNC and ESC did not result in inferior survival and should be applied to patients with a poor prognosis. Esophagectomy correlates with good outcomes and should be encouraged if the patient has a good prognosis.

## Introduction

Head and neck cancer (HNC) and esophageal cancer (ESC) usually share a common aetiology. It is highly possible for these two cancers to develop in one patient.^1,2^ In recent years, staging and detection tools have become more powerful, and synchronous cancers are frequently detected after a systemic survey.^3,4^ When these two cancers are in their early stages, local therapy is well-tolerated and effective; however, when both cancers occur synchronously and one of the cancers is advanced, treatment becomes complicated. Treatment for HNC and ESC usually requires combining surgery, radiotherapy, and chemotherapy.^5–8^ Each of these treatments leads to moderate to severe adverse events in patients. Furthermore, patients with HNC and ESC usually present with malnutrition before treatment.^9,10^ Thus, the adverse events are usually intolerable and treating these two cancers safely and effectively is difficult. The treatment results of available studies have also showed poor overall survival (OS) rates.^11–13^ Currently, patients with synchronous HNC and ESC are treated with different modalities in clinical practice, and a standard treatment protocol is not available. To improve treatment outcomes, a safe and effective treatment modality should be identified. For advanced esophageal cancer, a split-course treatment has been applied in some cases to reduce the risk of severe complications.^14^ In our hospital, we adopted the same concept to treat synchronous HNC and ESC in a split course. This retrospective study was designed to review the results of different treatment modalities for patients who have synchronous HNC and ESC. We would like to exam the effectiveness of protracted course treatment and to identify prognostic factors that can help us to improve the treatment outcome.

## Methods

With the permission of the institutional review board, we retrieved clinical data for HNC and ESC patients from a cancer registry in May 2015. Advanced stage was defined as a cancer stage that indicated how chemoradiation was a part of treatment. As such, the following inclusion criteria were employed: both cancers were found within 30 days; there was a documented pathology of invasive cancer; and either one of the cancers were in their advanced stages that chemoradiation was indicated (HNC stage III/IV or ESC stage II or higher; as determined by the American Joint Committee on Cancer staging system, seventh edition).^15^ Patients were excluded for the following reasons: evident metastatic disease, Eastern Cooperative Oncology Group (ECOG) three or 4, a history of prior cancer, or palliative treatment assigned by any means. According to the above criteria, 51 patients were selected between May 2004 to April 2012. All data were reviewed after retrieval. Tumor staging was revised according to the seventh edition of American Joint Committee on Cancer staging system. Patient characteristics, disease status, and treatment parameters were recorded, but personally identifying information was erased before analysis.

Treatment greatly varied between patients. Radical surgery was the primary treatment for oral cavity cancer and typically involved wide excision and neck dissection. For pharyngeal and laryngeal cancer, laser excision and chemoradiation (CRT) were the primary treatments for stages I–II and other disease stages, respectively. Preoperative CRT and radical surgery were recommended for ESC, but esophagectomy was not performed in every patient due to patient preference. Three different treatment methods were classified according to the sequence of CRT for HNC and ESC:

1. A continuous CRT course irradiating the two cancers at the same time (c-CRT);
2. A protracted CRT course irradiating HNC and ESC separately or with intended interruptions (p-CRT); and
3. Induction chemotherapy followed by local treatment (IC).

Radiotherapy consisted of prophylactic irradiation for regional lymphatics with a boost to high-risk areas or gross tumor and lymph nodes. The doses for HNC for prophylactic irradiation, high-risk areas, and gross tumor/lymph nodes were 46–50 Gy, 60–66 Gy, and 72 Gy, respectively. The doses for ESC for prophylactic irradiation and gross tumor/lymph nodes were 30–36 Gy and 50–60 Gy, respectively. The fraction size was 1.8 or 2 Gy with a schedule of 1 fraction per day, 5 days per week. For c-CRT group, the treatment should be completed in 56 days. For p-CRT group, radiotherapy for HNC and ESC should be completed in 56 days and 35–42 days, respectively. But the interval for rest between two treatment was or any interruption during RT would be judged by physician’s discretion. For HNC, intensity-modulated radiotherapy was used. For ESC, intensity-modulated radiotherapy was used alone or in combination with an anteroposterior opposing field with three-dimensional dose calculation. All patients received chemotherapy, which typically consisted of cisplatin and 5-fluorouracil (5-FU) or 5-FU prodrug. Induction chemotherapy was administered to some patients and included various combinations of cisplatin, 5-FU, and docetaxel.^16–19^

Outcome measures included tumor progression and death, with each tumor progression and recurrence recorded as tumor progression. Response evaluations were performed 3 months following treatment completion and included computed tomography scan or magnetic resonance imaging, esophagogastroduodenoscopy, and esophagogram. All patients were followed up at an outpatient department every 3–4 months for the first 3 years after treatment, and every 6–12 months thereafter. During follow up, imaging and endoscopic examinations were arranged annually, at a minimum, or when signs of progression were observed. Re-staging studies in patients with recurrent tumors or second primary cancers were used to define tumor extent. Optimal treatment or supportive care was given depending on the status of the disease and the patient. The cause of death and failure patterns were reviewed if any evidence of tumor progression was observed. The cause of death was recorded as “disease” until every suspicion was ruled out. Progression was verified by pathological examination or consequent clinical findings. The date of progression was recorded with the day of the first note in the chart, indicating signs of possible progression. Second, primary cancers and deaths unrelated to progression or adverse events were not considered treatment failure. The primary endpoint was OS and the secondary endpoint was progression-free survival (PFS). The period of survival was calculated from the date of first pathologic diagnosis to the date of either tumor progression or “death from disease” for PFS, or death for OS.

All characteristics and treatment parameters were categorized according to available references. Anemia was defined as a hemoglobin level <13 g dl^−1^.^20^ We used the Kaplan–Meier method for survival analysis and log-rank tests to determine whether there were significant differences between the patients in terms of primary and secondary endpoints. Multivariate analysis was performed using a Cox regression model to assess the ability of prognostic factors to predict survival outcomes (expressed as an odds ratio and 95% confidence interval). The correlation of each variable to each endpoint was evaluated by both univariate and multivariate analyses. Differences were considered significant when the *p*-value was <0.05. We used the commercial statistical package, PASW Statistics 18.0, to perform all statistical analyses (SPSS Inc. Chicago, IL).

## Results

### Patient population

A total of 51 patients were included in this study and their ages ranged from 33 to 76 years old, with a median of 53 years. All patients were males. Most patients had a diagnosis of hypopharyngeal cancer for HNC (*n* = 27; 52.9%), followed by oropharyngeal cancer (*n* = 14; 27.5%), laryngeal cancer (*n* = 8; 15.7%), and oral cavity cancer (*n* = 2; 3.9%). The clinical stages of HNC were I, II, III, and IV in 4 (7.8%), 6 (11.8%), 9 (17.5%), and 32 (62.7%) patients, respectively. The clinical stages of ESC were I, II, and III in 14 (27.5%), 13 (25.4%), and 24 (47.1%) patients, respectively. All Stage IV ESC patients were excluded since palliative treatment was recommended due to their poor prognosis and performance status. The median follow-up period was 29 months for living patients (range: 24–81 months). Other characteristics are listed in Table 1.

**Table 1.** Characteristics and treatment parameters of all patients

**Characteristic**	**Frequency (%)**
Age (years)	Median: 53 (33–76)
Sex	
Male	51 (100%)
Female	0 (0.0%)
Marital status	
Single	5 (9.8%)
Married	42 (82.4%)
Divorced	4 (7.8%)
ECOG performance status	
0–1	44 (92.2%)
2	7 (7.8%)
Feeding tube	
No	41 (80.4%)
Yes	10 (19.6%)
Tracheostomy	
No	49 (96.1%)
Yes	2 (3.9%)
Smoking	
No	1 (1.9%)
Yes	50 (98.1%)
Alcohol consumption	
No	5 (8.9%)
Yes	46 (91.1%)
Betel quid chewing	
No	17 (33.3%)
Yes	34 (66.7%)
Other systemic diseases	
Denied	35 (68.6%)
Yes	16 (31.4%)
Anaemia (<13 g dl^−1^)	
No	26 (51.0%)
Yes	25 (49.0%)
HNC location	
Larynx and hypopharynx	35 (68.6%)
Oral cavity and oropharynx	16 (31.4%)
HNC histology	
Well and moderate differentiation	43 (84.3%)
Poor differentiation	8 (15.7%)
HNC clinical stage	
I	4 (7.8%)
II	6 (11.8%)
III	9 (17.7%)
IV	32 (62.7%)
ESC histology	
Well and moderate differentiation	40 (78.4%)
Poor differentiation	11 (21.6%)
ESC location	
Cervical/upper third	11 (21.6%)
Middle third/lower third	40 (78.4%)
ESC clinical stage	
I	14 (27.5%)
II	13 (25.4%)
III	24 (47.1%)
Advanced cancer status	
HNC (stage III or IV)	14 (27.5%)
ESC (stage II–IV)	10 (19.6%)
Both	27 (52.9%)
Treatment method	
c-CRT	28 (54.9%)
p-CRT	11 (21.6%)
IC	12 (23.5%)
Surgery for HNC	
No	39 (76.5%)
Yes	12 (23.5%)
Surgery for ESC	
No	35 (68.6%)
Yes	16 (31.4%)
Interval between treatments	
30 days or less	35 (68.6%)
More than 30 days	10 (19.6%)
*Others	6 (11.8%)
Total treatment duration	
90 days or less	25 (49.0%)
More than 90 days	18 (35.3%)
Incomplete	8 (15.7%)

ESC, esophageal cancer; HNC, head and neck cancer.

a Others: Either only one cancer was treated and then disease progression was observed, or the patient did not complete the planned treatment.

### Treatment

**Figure 1.**
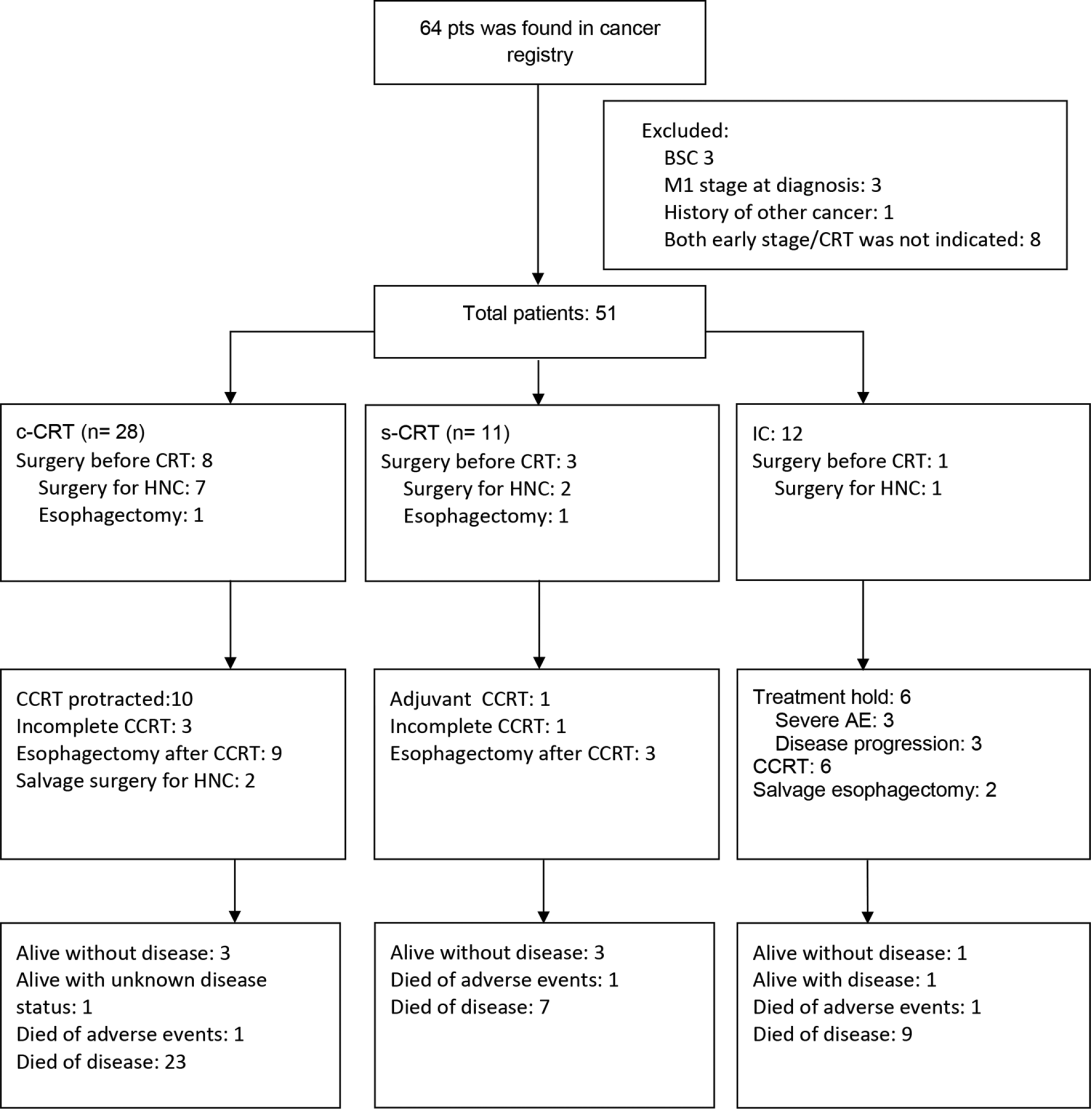
Treatment course of all patients.

There were 28 (54.9%), 11 (21.6%), and 12 (23.5%) patients treated by c-CRT, p-CRT, and IC, respectively. The treatment courses of each patients were demonstrated in Figure 1. 8 of 12 patients in the IC group received induction chemotherapy via a docetaxel-based regimen, while the other four received induction chemotherapy with a cisplatin-based regimen. 12 patients received primary surgery for HNC and 16 received esophagectomy for ESC. The distribution of esophagectomy in different clinical stage of ESC was similar. Seven of 27 clinical Stage I–II ESC and 9 of 24 clinical Stage III ESC received esophagectomy (significance: 0.546, Fisher’s exact test, 2-sided). Treatment was not completed for 2, 1, and 6 patients in the c-CRT, p-CRT, and IC groups, respectively. The frequency of incomplete treatment was significantly higher in the IC group (50%) than in the c-CRT (5%) and p-CRT (10%) groups (one-way analysis of variance [ANOVA], *p* < 0.01). Three of the eight patients who received a docetaxel-based regimen did not complete treatment due to adverse events. Additionally, three of the four patients who received a cisplatin-based regimen did not complete treatment due to disease progression. Three patients (one in each treatment group) died of treatment-related infections. The interval between treatments for HNC and ESC ranged from 0 to 120 days (median: 0 days). The total treatment duration ranged from 31 to 380 days (median: 82 days). The median treatment durations were 57, 148, and 92 days for the c-CRT, p-CRT, and IC group, respectively. 7 of 19 patients in the c-CRT group who completed the entire course of treatment still had their RT courses protracted. Table 1 lists the treatment parameters.

### Overall survival

**Figure 2.**
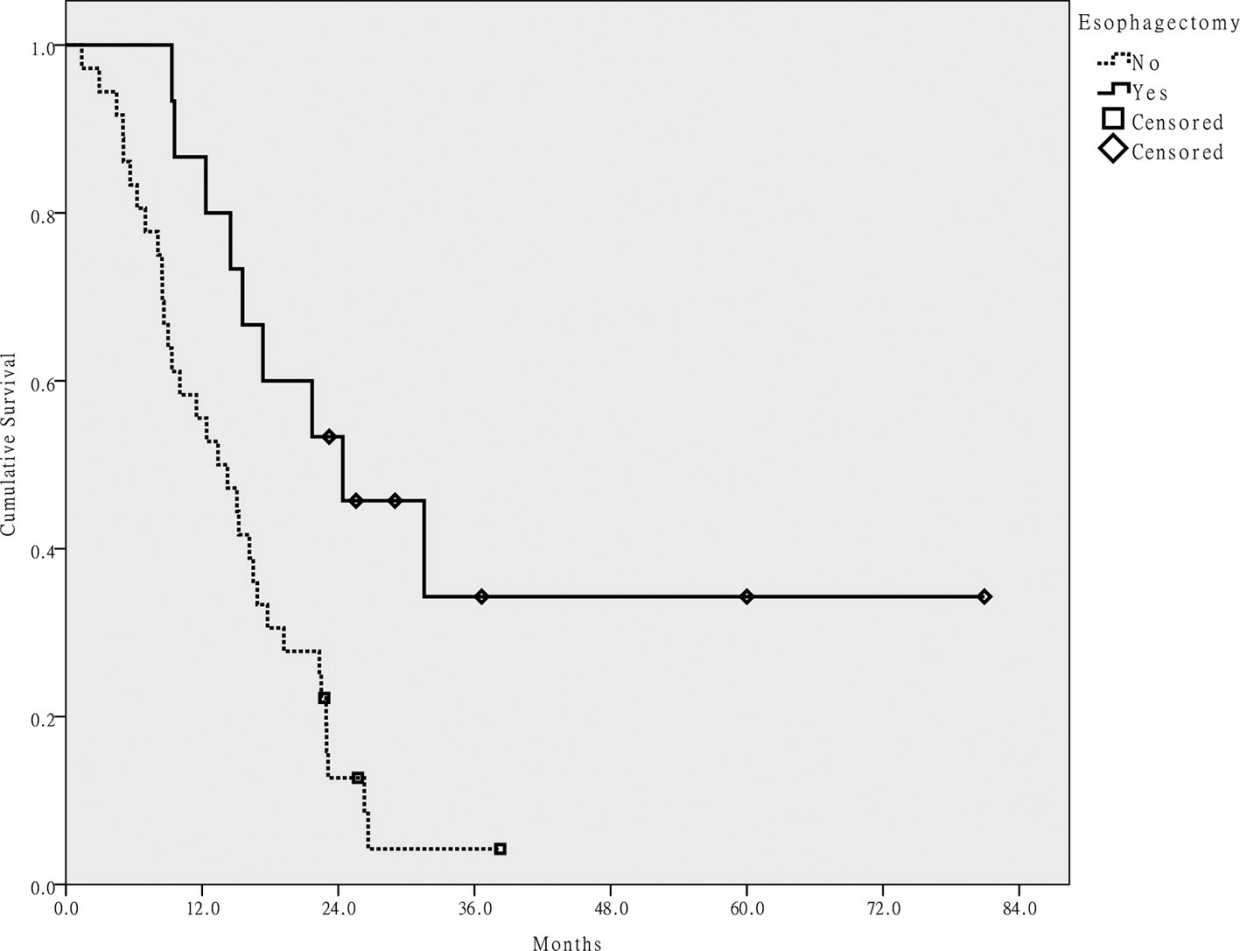
Overall survival of patients who did or did not receive a primary esophagectomy.

At the time of analysis, 42 of 51 patients had died. The median follow-up period was 29 months for living patients (range: 24–81 months). The cause of death was cancer progression in 39 cases and treatment-related adverse events in 3. At the time of analysis, seven patients were alive without disease, one patient was alive with disease, and one patient was alive with unknown disease status. The 2 year OS rate of all patients was 25.1% and the median survival was 15 months. Via univariate analysis, anemia, HNC located in the larynx or hypopharynx, and no esophagectomy correlated with poor OS (*p* < 0.05). On multivariate analysis, anemia and no esophagectomy were poor independent prognostic factors (*p* < 0.05; Table 2). Figure 2 shows the OS in patients with or without esophagectomy.

**Table 2.** Multivariate Cox regression analysis – impact on overall survival

**Characteristics**	**2 Year Overall Survival**	**Multivariate Analysis**	**Multivariate Analysis**
		**Significance**	**Hazard Ratio (95% CI)**
Anaemia			
Yes	15%	0.037	1.94 (1.04–3.616)
No	33.80%		
Esophagectomy			
No	13.10%	0.008	2.811 (1.315–6.01)
Yes	50%		

### Progression-free survival

**Figure 3.**
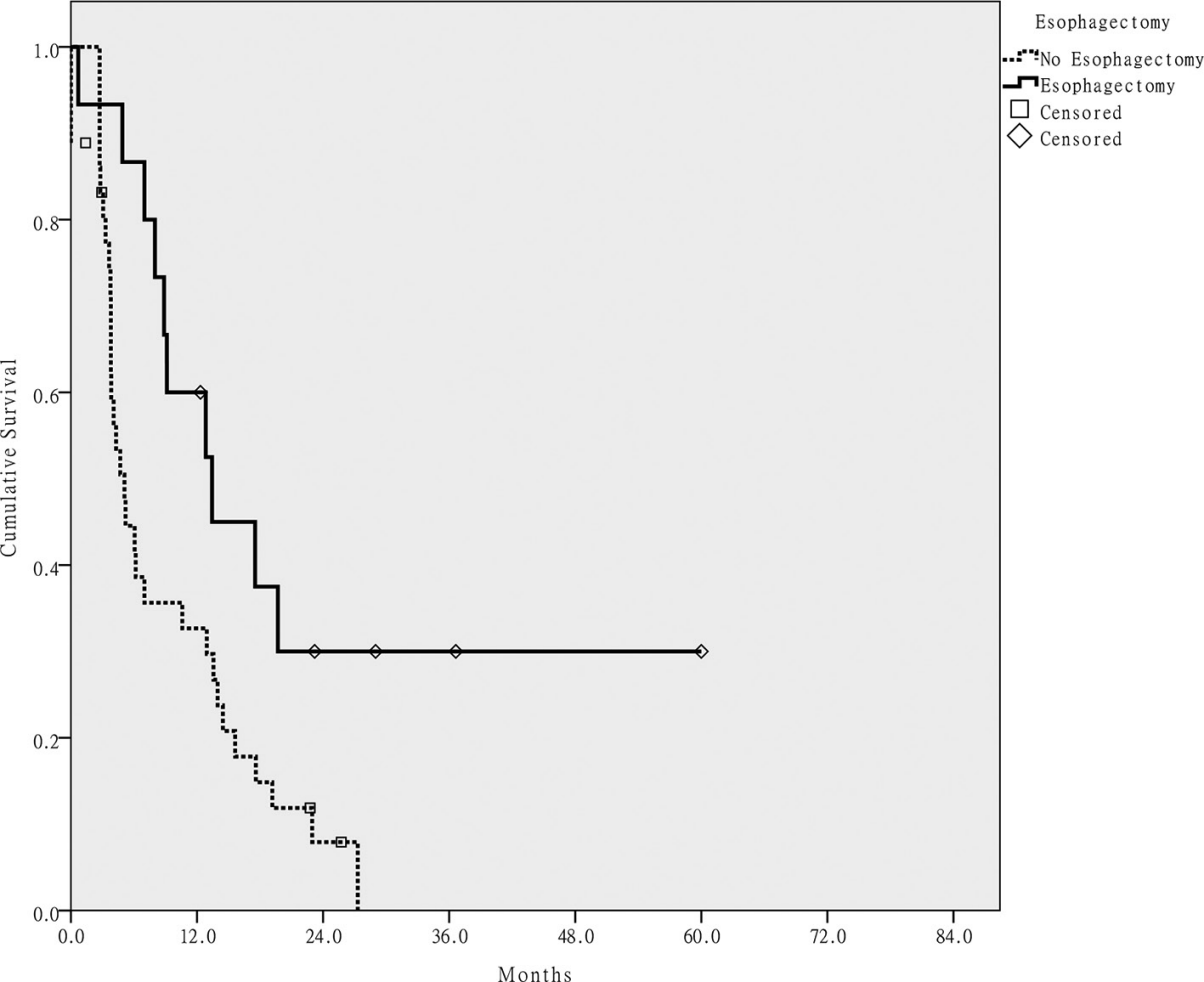
Progression-free survival of patients who did or did not receive a primary esophagectomy.

42 patients had documented disease progression. The 2 year PFS rate for all patients was 14.8%. Three patients died of treatment-related adverse events; therefore, the treatment response of both cancers was not evaluated. Five patients had no evaluation for ESC due to HNC progression. HNC was controlled in 22 patients and ESC was controlled in 17 patients. Only two cases of cancer progression were salvaged by radical surgery. On univariate analysis, only no esophagectomy correlated with poor PFS (*p* < 0.05). Figure 3 shows the PFS of patients who did or did not receive esophagectomy. In patients who did not received esophagectomy, 18 of 35 patients experienced local recurrence. But only 5 of 15 patients who received esophagectomy had local recurrence (χ^2^ test, *p* = 0.106).

## Discussion

Treating either advanced HNC or ESC is difficult. When these two diseases co-occur, this difficulty is enormously increased, and the treatment outcomes are usually poor. The available clinical results showed a 2 year OS of around 40%, with some patients with both cancers in their early stages.^11–13^ The current study showed results that were inferior to others, since patients were excluded if both cancers were in their early stages. We also found that p-CRT was not inferior to c-CRT in terms of OS and PFS. A protracted treatment would not deteriorate treatment outcomes. Esophagectomy was the most important prognostic factor which associated with better OS and PFS.

In general, the schedule of curative treatment is continuous, as it can avoid tumor cell repopulation. A split or protracted treatment course can allow the cancer cells to repopulate, increasing the risk of tumor progression. However, tumor control is not the only factor that correlates with OS. Some studies have shown that even with exceptional tumor control, the improvement in OS is not significant.^21,22^ Adverse events, second primary cancers, and distant metastasis may negate the benefit of improved tumor control. In a randomized trial of CRT for esophageal cancer using both continuous and split radiotherapy courses, the split radiotherapy courses did not correlate with inferior survival.^14^ In the current study, 37% of patients in the c-CRT group had their treatment course protracted unintentionally. This implies that c-CRT is not tolerable for patients who have synchronous HNC and ESC. Most patients with HNC and ESC have a poor nutrition status before treatment, which is correlated with poor compliance of toxic treatment.^6,8^ The treatment volume of c-CRT may cover nearly the entire esophagus and pharynx, which can cause severe adverse events. Therefore, a slightly protracted course may be more reasonable for patients with synchronous HNC and ESC.

Induction chemotherapy is frequently used to improve the treatment results of various cancers. However, the current study showed that a cisplatin-based regimen was not effective. Three of the four patients had disease progression during cisplatin-based induction chemotherapy, and all four patients died of disease progression. In literature, the response rate of cisplatin-based induction chemotherapy for squamous cell carcinoma of the esophagus ranges from 40 to 60%.^23–26^ Further, cisplatin-based chemotherapy leads to a response rate in HNC of approximately 50–80%.^27,28^ As such, the chance of achieving an obvious response at both sites will be <50%. Taxane-based chemotherapy was more effective for both HNC and ESC, consistent with prior clinical trial findings.^19,29–31^ However, taxane-based chemotherapy appeared to be too toxic for patients with synchronous HNC and ESC. In the present study, three of the eight patients who received taxane-based chemotherapy ceased treatment due to critical adverse events.

For ESC with squamous cell type, definitive chemoradiotherapy provides low but certain rate of long-term survival, which is not inferior to preoperative chemoradiotherapy followed by surgery. Two randomized trials directly comparing chemoradiotherapy alone with trimodality therapy (chemoradiotherapy followed by surgery) have failed to demonstrate better survival, although both show better locoregional control and a lesser need for palliative procedures when surgery is a component of multimodality treatment.^14,32^ However, the current study showed that esophagectomy correlated with both better OS and tumor control. The 2 year OS rate and PFS rate were extremely low when esophagectomy was not performed. The percentage of clinical Stage III ESC were similar in patients who received esophagectomy and who did not. The distribution of esophagectomy in different response group after chemoradiation was also reviewed. Esophagectomy was performed most commonly in patients who had partial remission (7/14) and progressive disease (3/7), followed by complete remission (4/15) and stable disease (2/7). One patient received esophagectomy without prior chemoradiation and six patients did not go on treatment because of toxicities and events related with HNC. Though selection bias could not be eliminated totally, the prognostic effect of esophagectomy was not severely biased by selection. So esophagectomy has an important role in treatment of synchronous HNC and ESC.

Maybe there are some important factors that affect the tumor control and OS in this small sample size and retrospective study. We discuss the only significant factors after statistic analysis to focus the study result and avoid too long length of article.

## Conclusion

The outcomes are poor for patients with advanced synchronous HNC and ESC. Esophagectomy correlated with good prognosis and should be performed for patients if possible. Radiotherapy with a split or protracted course does not result in inferior treatment result and can be considered when the aim is to avoid adverse events. Clinical trials based on the available evidence should be conducted and combining with novel medicine and technique to find a better treatment method for these patients.
